# Application status and research progress of targeted therapy drugs for hormone receptor-positive breast cancer

**DOI:** 10.3389/fmed.2025.1513836

**Published:** 2025-03-11

**Authors:** Han Luo, Yue Sun, Tiefeng Xu

**Affiliations:** ^1^Department of Breast Surgery, Hainan Affiliated Hospital of Hainan Medical University (Hainan General Hospital), Haikou, China; ^2^Department of Breast Surgery, The First Affiliated Hospital of Hainan Medical University, Haikou, China

**Keywords:** hormone receptor-positive breast cancer, targeted therapy, precision medicine, female health, clinical trials

## Abstract

Breast cancer (BC) is the most common malignant tumor in women and the leading cause of cancer-related deaths in women. As one of the most common subtypes of breast cancer, patients with hormone receptor-positive (HR+) breast cancer usually experience disease progression over an extended period of time, triggering the search for therapeutic strategies other than endocrine therapy. In recent years, continuous research on various targets has led to dramatic changes in the treatment of hormone receptor-positive breast cancer patients, resulting in prolonged clinical survival. With the redefinition of human epidermal growth factor-2 (HER2) expression, more precise and individualized treatment is possible. This review comprehensively reviews targeted therapies and critical clinical trials for HR+ breast cancer and tracks the latest advances. It also provides valuable insights into the future direction of targeted therapies.

## 1 Introduction

The most prevalent malignant tumor in women worldwide is breast cancer (BC), which has a significant impact on both the physical and emotional health of women (1). Molecular typing is essential in tumor treatment since breast cancer is extremely diverse, and treatment outcomes differ from person to person. The 2013 St. Gallen International Breast Cancer Conference guidelines introduced the widely recognized molecular typing, which divides breast cancer into four subtypes: basal-like, Luminal A, Luminal B, and overexpression of human epidermal growth factor-2 (HER2) (2). Triple-negative breast cancer accounts for about 10–15% of breast cancers, HER2-positive (HER2+) breast cancer accounts for 15–20% of breast cancers, and hormone receptor-positive (HR+) breast cancer (covering Luminal A and Luminal B types) is the most common subtype, accounting for about 70–75% of all breast cancer cases. It is widely acknowledged that excess estrogen triggers the expression of target genes in estrogen receptor-positive breast cancer, resulting in the development of estrogen-stimulated breast cancer (3–5).

The use of endocrine drugs is crucial for the treatment of HR+ breast cancer, inhibiting tumor recurrence, proliferation, and metastasis by antagonizing estrogen. Currently, endocrine drugs mainly include selective estrogen receptor modulators (SERMs), aromatase inhibitors (AIs), selective estrogen receptor down regulators (SERDs), luteinizing hormone-releasing hormone (LHRH) analogs, and progesterone (1, 6, 7). Despite the better prognosis of endocrine therapy for HR+ breast cancer, the 5-year survival rate is still below 30% (8). Extended endocrine therapy alone in patients with an intermediate to high risk of recurrence does not meet the clinical needs, and patients often progress and metastasize. Therefore, new treatment options must be explored. As precision therapy continues to progress, targeted agents, including cyclin-dependent kinase 4 and 6 inhibitors (CDK4/6is), PI3K/AKT/mTOR(PAM) inhibitors, HER2 inhibitors, vascular endothelial growth factor (VEGF) inhibitors, and histone deacetylase inhibitors (HDACis), are being combined with endocrine drug therapy to improve tumor prognosis.

This review summarizes the advances in targeted drug therapy for HR+ breast cancer, systematically reviews the relevant major clinical studies, and discusses future directions.

## 2 Targeting Cyclin D-CDK4/6-Rb pathway

### 2.1 Cyclin D-CDK4/6-Rb pathway in HR+ breast cancer

**Figure 1:**
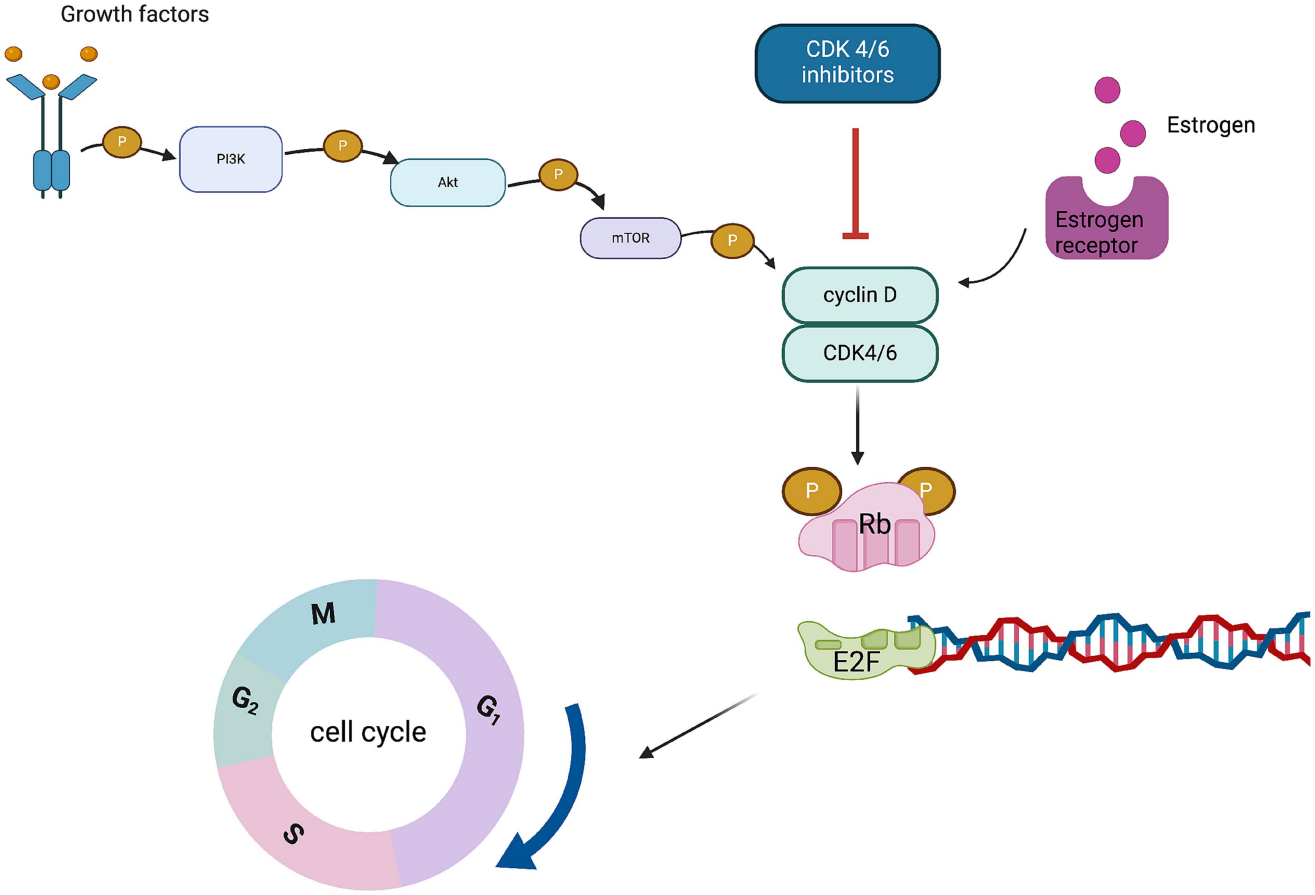
Mechanism of action of cyclin-dependent kinase 4/6 inhibitors.

One of the leading causes of tumor growth and medication resistance in breast cancer is cell cycle disruption. CDK4/6 is a sensor that links multiple signaling pathways to cell cycle initiation and progression (9, 10). By encouraging the phosphorylation of retinoblastoma protein (Rb), which releases the transcription factor E2F and stimulates gene transcription from the G to S phase, CDK4/6 stimulates cell proliferation. In HR+ breast cancer, the Cyclin D-CDK4/6 signaling pathway activated by the estrogen pathway is an important cause of tumor proliferation and endocrine therapy resistance (11). Anti-tumor effects can be achieved by inhibiting CDK4/6, which stops cells in the G1 phase and prevents cell mitosis (12) (Figure 1).

### 2.2 CDK 4/6 inhibitors

Palbociclib, ribociclib, and abemaciclib are the three FDA-approved CDK4/6is for breast cancer, which are currently the first- and second-line systemic treatment choices for HR+/human epidermal growth factor-2 negetive (HER2-) advanced/metastatic BC. These drugs can be used alone or in conjunction with endocrine therapy (13). Additionally, CDK4/6is has demonstrated remarkable clinical effects in neoadjuvant endocrine therapy and early breast cancer, as evidence continues to grow and the therapeutic range of CDK4/6 inhibitors continues to broaden. Clinical trials are underway for new CDK4/6is, like dalpiciclib.

#### 2.2.1 CDK4/6 inhibitors in advanced/metastatic HR+/HER2- breast cancer

Advanced breast cancer refers to the status of locally advanced inoperable or cancer cells metastasizing to distant sites through blood vessels, lymphatic channels, etc., which usually indicates a poor prognosis. Locally advanced breast cancer can gain access to surgery through neoadjuvant chemotherapy. Surgery and radiotherapy for metastatic breast cancer are usually not the first choice, and patients are unable to achieve a cure; instead, the goal is to prolong the patient’s clinical survival and reduce drug toxicities. In patients with HR+ breast cancer, the risk of distant metastatic recurrence is higher in patients treated for more than 5 years than in other subtypes and rises progressively over time (14).

As the first CDK4/6i approved by the FDA for use in combination with endocrine therapy in patients with HR+/HER2- advanced breast cancer (ABC), palbociclib demonstrated impressive therapeutic efficacy in the PALOMA-1/TRIO-18 phase II clinical trial. Its combination with letrozole in postmenopausal patients with HR+/ HER2- ABC resulted in a 10-month improvement in median progression-free survival (mPFS) compared to the control group. Still, there was no statistically significant improvement in the subsequent analysis of median overall survival (mOS) (15). The subsequent phase III PALOMA-2 trial further validated the clinical efficacy of palbociclib. However, it again did not significantly improve mOS after completing the final follow-up visit (16, 17). The therapeutic interval for palbociclib was further broadened by PALOMA-3, which explored the efficacy of palbociclib in patients with HR+/HER2- ABC that progressed after endocrine therapy(ET) and showed that the addition of palbociclib to fulvestrant prolonged mPFS by 4.9 months compared to fulvestrant alone, and achieved a significant prolongation of mOS at median significant prolongation in mOS was obtained after 73.3 months of follow-up [hazard ratio (HR), 0.81; 95% confidence interval (CI), 0.65–0.99] (18, 19). PALOMA-4 focused on the Asian population and showed that palbociclib in combination with letrozole prolonged mPFS in previously untreated HR+/HER2- ABC postmenopausal Asian women (21.5 months vs. 13.9 months; HR, 0.68; 95% CI, 0.53–0.87; *p* = 0.0012) (20). As a pioneering CDK4/6i, it has shown impressive clinical benefit in the treatment of HR+ ABC, transforming the treatment regimen for HR+/HER2- ABC.

Rebociclib, another small molecule selective for CDK4/6i, has a large binding site and substituent that avoids binding to CDKs other than CDK4/6. Compared to palbociclib and abemaciclib, ribociclib has a higher drug binding rate (21). In the phase III MONALEESA⁃2 study, ribociclib in combination with letrozole prolonged mPFS in previously untreated postmenopausal HR+/HER2- metastatic breast cancer (MBC) patients (25.3 months vs. 16.0 months; HR, 0.57; 95%Cl, 0.46–0.70) (22). MONALEESA-3 explored efficacy in postmenopausal HR+/HER2- MBC patients who had progressed after prior endocrine therapy and showed that ribociclib in combination with fulvestrant significantly improved progression-free survival (PFS) compared to control (20.5 months vs. 12.8 months; HR, 0.59; 95% CI, 0.59; 95% CI, 0.48–0.73; *p* < 0.001), and the use of ribociclib resulted in an overall survival (OS) benefit after extended follow-up (mPFS 56.3 months) (23). The MONALEESA-7 study investigated the effectiveness of ribociclib in premenopausal and perimenopausal patients with HR+/HER2- ABC. The results showed that ribociclib significantly improved mPFS (23.8 vs. 13.0 months; HR, 0.55; 95% CI, 0.44–0.69; *p* < 0.001). The benefit was also demonstrated in OS through prolonged follow-up (median 53.5 months), with an OS of 58.7 months for ribociclib combined with ET versus 48.0 months for the placebo group (HR, 0.76; 95% CI, 0.61–0.96) (24). This study is noteworthy as it is the first clinical trial to utilize CDK4/6is in treating premenopausal breast cancer patients, offering a new treatment option for those with advanced disease.

Abemaciclib is the third CDK4/6 inhibitor approved for marketing by the FDA, following palbociclib and ribociclib. In the MONARCH 1 trial, abemaciclib monotherapy demonstrated statistically and clinically significant efficacy in treating patients with HR+ ABC or MBC, showing a mPFS of 6.0 months and a mOS of 17.7 months. Currently, these are the only FDA-approved CDK4/6is for the treatment of ABC or MBC (25). In the MONARCH-2 trial, abciximab combined with fulvestrant increased mPFS by 7.1 months (HR, 0.553; 95% CI, 0.449 to 0.681; *p* < 0.001), with a significant improvement in OS, in HR+/HER2- MBC patients with prior ET vs. placebo combined with fulvestrant (46.7 months vs. 37.3 months; HR, 0.76; 95% CI, 0.61–0.95; *p* = 0.01) (26). The MONARCH-3 study explored the efficacy of abemaciclib in combination with letrozole or anastrozole in patients with hormone HR+/HER2-, perimenopausal ABC who had not received systemic therapy. After 8.1 years of follow-up, the results showed that PFS was significantly prolonged for the group receiving abemaciclib compared to the placebo group (29.0 months versus 14.8 months; HR, 0.693; 95% CI 0.557–0.863; *p* = 0.0010). Additionally, the OS analysis indicated that the mOS for patients who received abemaciclib increased by 13.1 months, resulting in a mOS of 66.8 months compared to 53.7 months in the placebo group. However, the final analysis did not achieve statistical significance (27). The results indicated that the combination of abemaciclib and tamoxifen achieved a PFS benefit of more longer months compared to abemaciclib alone; however, this difference was not statistically significant (28).

Dalpiciclib, a novel CDK4/6 inhibitor, showed a significant increase in mPFS for the fluvastatin and dalpiciclib combination in HR+/HER2- ABC patients in the DAWNA-1 trial (15 months vs. 7.2 months; HR, 0.42; 95% CI, 0.31–0.58) (29). The DAWNA-2 clinical study examined the combination of dalpiciclib with anastrozole or letrozole for the treatment of postmenopausal HR+/HER2- ABC in previously untreated patients. Results showed a benefit of dalpiciclib in mPFS (30.6 months vs. 18.2 months; HR, 0.51; 95% CI, 0.38–0.69) (30). While more experimental data on these combinations are currently being explored, dalpiciclib is anticipated to become a promising option for future therapies that integrate CDK4/6is with ET.

#### 2.2.2 CDK 4/6 inhibitors in early HR+/HER2- breast Cancer

The efficacy of CDK4/6is in the adjuvant treatment of HR+/HER2- ABC has been established, but in the clinic, more than 90% of breast cancer patients are diagnosed at an early stage (31). However, there is still a high risk of recurrence for patients at high risk. Therefore, clinical studies have been conducted to investigate whether CDK4/6 inhibitors can provide clinical benefit to patients with early-stage, high-risk breast cancer through intensive adjuvant therapy. The PALLAS study demonstrated that the addition of palbociclib to adjuvant endocrine therapy for 2 years did not improve invasive disease-free survival (iDFS) in HR+/HER2-early-stage breast cancer (eBC) (32). Results from the monarchE trial showed that the addition of 2 years of abemaciclib to endocrine therapy significantly improved iDFS in patients with HR+/HER2-, lymph node-positive, high-risk, early-stage breast cancer (33). The NATALEE study broadened the inclusion criteria to include patients with stage II or stage III, early-stage breast cancer, which include patients with lymph node-negative cancers, who also showed statistically significant improvements in iDFS, DDFS, and DRFS after 3 years of ribociclib combined with endocrine therapy compared to the control group (34).

#### 2.2.3 CDK 4/6 inhibitors in neoadjuvant endocrine therapy

HR+/HER2- BC patients are usually considered less sensitive to chemotherapy, and only about 10 to 20% of luminal-type patients can achieve pathological complete response (pCR) with neoadjuvant chemotherapy (35). Neoadjuvant endocrine therapy (NET) has gained attention as a possible alternative to neoadjuvant chemotherapy (NCT) as a reasonable and feasible treatment strategy for HR+ patients (36). A meta-study showed that NET did not significantly differ from NCT regarding clinical remission rate, radiologic remission rate, and BCS rate in HR+ BC patients (37). As a result, a series of clinical studies have been conducted to explore whether the addition of CDK4/6is to NET can improve the prognosis of patients. According to the NeoPalAna study, the rate of full cell-cycle arrest was higher when palbociclib was added to anastrozole for NET in HR+/HER2- BC patients than when ET was used alone. In the CORALLEEN trial, NET with ribociclib plus letrozole in HR+/HER2-, eBC resulted in higher rates of complete cell cycle arrest than chemotherapy alone. Abciximab combined with anastrozole in the neoMONARCH trial achieved a significant reduction in Ki67 expression after 2 weeks of neoadjuvant therapy in patients with HR+/HER2- BC. It resulted in cell cycle arrest (38–40).

#### 2.2.4 New explorations of CDK 4/6 inhibitors

CDK4/6is has been effective in treating HR+ BC, greatly improving the prognosis of patients. Clinical benefit has been seen in both early adjuvant therapy and NET. Thus, the efficacy and safety of FDA-approved CDK4/6is in ABC have been more extensively explored. The MAINTAIN phase II clinical trial demonstrated a prolonged mPFS in patients with HR+/HER2- MBC with the addition of ribociclib during switching to a different ET agent (5.29 months vs. 2.76 months; HR, 0.57; 95% CI, 0.39–0.85; *p* = 0.006) (41). The postMONARCH study was the first to investigate whether switching to CDK4/6is could be beneficial for patients with HR+/HER2- ABC who experienced disease progression despite prior CDK4/6is therapy. The results showed that switching to abemaciclib in patients who had progressed on prior CDK4/6is boosted mPFS from 5.3 months to 6.0 months, a statistically significant difference that enriches the clinical data on cross-line therapy with CDK 4/6 inhibitors (42) Additionally, the PALMARES-2 study, a multicenter real-world investigation, was the first to compare the efficacy of palbociclib, ribociclib, and abemaciclib as first-line treatments for HR+/HER2- ABC. It showed that ribociclib and abemaciclib were independently associated with better real-world progression-free survival (rwPFS) compared to palbociclib, particularly in patients who were endocrine-resistant, had luminal B subtype tumors, or were premenopausal. For patients with *de novo* metastatic disease, abemaciclib was the most effective CDK4/6i, while ribociclib was more effective than palbociclib in patients with liver metastases. The three CDK4/6is demonstrated similar rwPFS in elderly patients and patients with bone metastases. However, because this was a retrospective study, clinically relevant characteristics of patients treated with the three agents were unbalanced, and there was a lack of mature OS data for comparison (Table 1).

**Table 1:** Clinical trial studies of CDK4/6i.

Drug	Trial	Stage	Programmatic	Patients/(numbers)	Dosage (mg)	Outcome			Reference
						mOS(months)	mPFS(months)	Others	
Palbociclib	PALOMA-1	II	palbociclib plus letrozole VS letrozole	Postmenopausal women with HR+/HER2- ABC (*n* = 165)	125 + 2.5 VS 2.5	37.5 VS 34.5	20.2 VS 10.2	–	15
	PALOMA-2	III	palbociclib plus letrozole VS letrozole	Postmenopausal women with HR+/HER2- ABC (*N* = 666)	125 + 2.5 VS 2.5	53.8 VS 49.8	27.6 VS 14.5	–	16,17
	PALOMA-3	III	palbociclib plus fulvestrant VS fulvestrant	HR+/HER2- ABC with ET before (*n* = 521)	125 + 500 VS 500	34.8 VS 28.0	9·5 VS 4·6	–	18,19
	PALOMA-4	III	palbociclib plus letrozole VS letrozole	Asian postmenopausal women with HR+/HER2- ABC (*n* = 340)	125 + 2.5 VS 2.5	–	21.5 VS 13.9	–	20
	PALLAS	III	palbociclib plus adjuvant ET VS adjuvant ET	HR+/HER2- eBC (*n* = 5,761)	125+ adjuvant ET 5 years VS adjuvant ET 5 years	not show any differences	not show any differences	Not improve iDFS	32
	NeoPalAna	II	palbociclib plus anastrozole VS anastrozole	Clinical stage II/III HR+/HER2- BC (*n* = 50)	125 + 1.0 VS 1.0	–	–	Improve CCCA rate significantly	38
Ribociclib	MONALEESA⁃2	III	ribociclib plus letrozole VS letrozole	HR+/HER2- ABC (*n* = 668)	600 + 2.5 VS 2.5	25.3 VS 16.0	–	–	22
	MONALEESA-3	III	ribociclib plus fulvestrant VS fulvestrant	HR+/HER2- ABC with ET before (*n* = 726)	600 + 500VS 500	53.7 VS 41.5	20.5 VS 12.8	–	23
	MONALEESA-7	III	ribociclib plus ET VS ET	HR+/HER2- ABC (*n* = 672)	600 + 1 VS 1	58.7 VS 48.0	23.8 VS 13.0	–	24
	NATALEE	III	ribociclib plus NSAI VS NSAI	HR+/HER2- eBC (*n* = 2,549)	400 + 2.5/1 VS 2.5/1	–	–	significant 25.2% relative reduction in iDFS and a significant improvement in DRFS	34
	CORALLEEN	III	ribociclib plus letrozole VS chemotherapy	HR+/HER2-, luminal BC postmenopausal women (*n* = 106)	600 + 2.5 VS chemotherapy	–	–	achieve molecular downstaging	39
	MAINTAIN	II	ribociclib plus ET VS ET	HR+/HER2- MBC with ET and CDK4/6i before (*n* = 119)	600 + 25/500 VS 25/500	–	5.29 VS 2.76	–	41
Abemaciclib	MONARCH 1	II	abemaciclib	HR+/HER2- MBC (*n* = 132)	200	17.7	6.0	–	25
	MONARCH-2	III	abemaciclib plus fulvestrant VS fulvestrant	HR+/HER2- MBC (*n* = 669)	150 + 500 VS 500	46.7 VS 37.3	16.4 VS 9.3	–	26
	MONARCH-3	III	abemaciclib plus NSAI VS NSAI	Postmenopausal women with HR+/HER2- ABC (*n* = 493)	150 + 1 / 2.5 VS 1 / 2.5	66.8 VS 53.7	29.0 VS 14.8	–	27
	NEXT MONARCH	II	abemaciclib plus chemotherapy VS abemaciclib	HR+/HER2- MBC with chemotherapybefore (*n* = 234)	150 + 20 VS 150/200	24.2 VS 17.0	9.1 VS 7.4	–	28
	monarchE	III	abemaciclib plus ET VS ET	HR+/HER2-, high-risk eBC (*n* = 501)	150 + ET VS ET	–	–	estimated 5-year IDFS rate: 85.9% vs. 79.1%; DRFS rate: 88.4% vs. 82.3%	33
	neoMONARCH	II	abemaciclib plus anastrozole VS abemaciclib VS anastrozole	HR+/HER2- BC (*n* = 224)	150 + 1 VS 150 VS 1	–	–	cell-cycle arrest 68% vs58%vs. 14%	40
	postMONARCH	III	abemaciclib plus fulvestrant	ABC following progression on CDK4/6 inhibition (*n* = 368)	150 + 500 VS 500	–	6.0 VS 5.3	–	42
Dalpiciclib	DAWNA⁃1	III	dalpiciclib plus fulvestrant VS fulvestrant	HR+/HER2- ABC with ET before (*n* = 361)		–	15.7 VS 7.2	–	29
	DAWNA⁃2	III	dalpiciclib plus ET VS ET	HR+/HER2- ABC (*n* = 456)	150 + 2·5 /1 VS 2·5 /1	–	30·6 VS 18·2	–	30

ABC, advanced breast cancer; MBC, metastatic breast cancer; eBC, early breast cancer; ET, endocrine therapy; DRFS, distant relapse-free survival; iDFS, invasive disease-free survival; CCCA, complete cell cycle arrest; NSAI, non-steroidal aromatase inhibitors.

## 3 Tgetting PI3K/AKT/mTOR pathway

### 3.1 PI3K/AKT/mTOR pathway in HR+ breast cancer

The phosphatidylinositol 3-kinase (PI3K)/protein kinase B (AKT)/mammalian target of rapamycin (mTOR) signaling pathway (PAM pathway) plays a pivotal role in breast carcinogenesis, with approximately 70% of breast tumors exhibiting hyperactivation of this pathway (43, 44). Activation of the pathway is initiated when ligands bind to receptor tyrosine kinases (RTKs) or G protein-coupled receptors (GPCRs) on the cell membrane, leading to the recruitment and activation of PI3K proteins. PI3K converts phosphatidylinositol 4,5-bisphosphate (PIP2) to phosphatidyl inositol 3,4,5-trisphosphate (PIP3), a second messenger that recruits and activates AKT. AKT, in turn, phosphorylates and activates downstream effectors such as pyruvate dehydrogenase kinase isozyme 1 (PDK1) and mammalian target of rapamycin complex 2 (mTORC2) at the plasma membrane. PDK1 and mTORC2 further activate AKT, which then phosphorylates and activates the mammalian target of rapamycin complex 1 (mTORC1). Activation of mTORC1 promotes protein and lipid synthesis via induction of ribosomal protein S6 (S6) and eukaryotic Translation Initiation Factor 4E-Binding Protein1 (4E-BP1) while reducing autophagy, ultimately driving cell growth and proliferation. The phosphatase and tensin homolog (PTEN) negatively regulates this pathway by converting PIP3 to PIP2. AKT phosphorylates and inactivates the negative regulator of mTORC1, TSC1/2, further activating this pathway (45–48).

**Figure 2:**
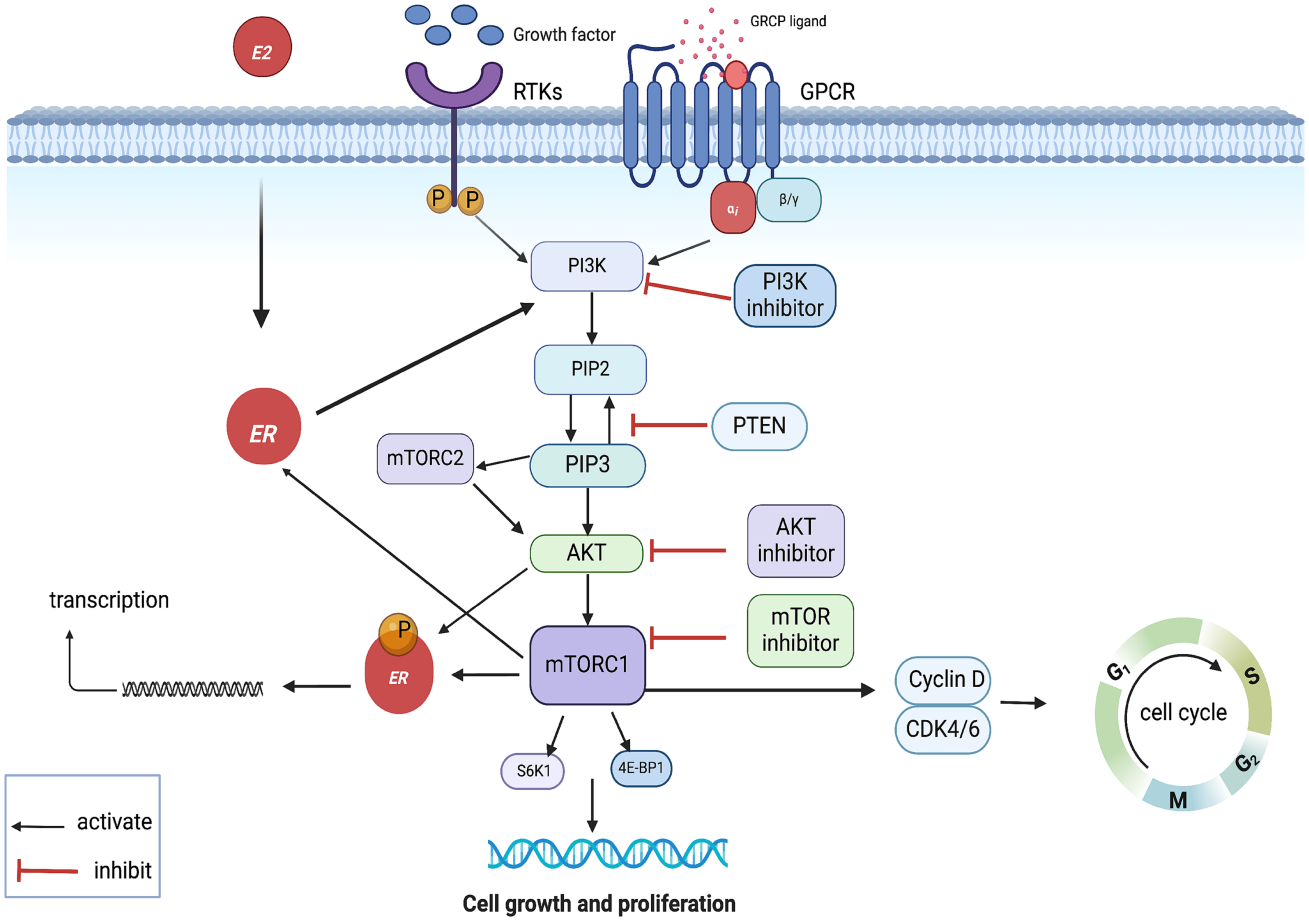
PAM pathway mechanism of action. GPCR, G protein- coupled receptors; RTKs, receptor tyrosine kinases; ER, estrogen receptor.

In HR+ BC, activation of the PI3K/AKT/mTOR pathway induces non-estrogen-dependent transcriptional activity through phosphorylation of estrogen receptor *α* (ERα) by AKT or mTOR. Similarly, ER activates the PI3K/AKT/mTOR pathway through activation of PI3K, which may be the reason for the ineffectiveness of endocrine therapy alone in some patients. This may be the reason for the poor outcome of endocrine therapy alone in some patients. In addition, it is worth noting that activation of the mutant PI3K/AKT/mTOR pathway in PI3K also promotes the progression of the cell cycle from G1 to S phase through downstream target activation of CDK4/6, which may attenuate the effect of CDK4/6is (Figure 2) (49, 50). Therefore, targeting the PAM pathway is an effective means to inhibit tumor proliferation.

### 3.2 PI3K/AKT/mTOR pathway inhibitors

Alpelisib is currently the only PI3K inhibitor approved for marketing by the FDA, and as an oral-specific PI3K kinase inhibitor, it inhibits PI3K better and has fewer side effects compared to pan-PI3K inhibitors (51). When treating PIK3CA-mutated HR+/HER2- ABC patients in the SOLAR-1 phase III clinical trial, apelalis plus fulvestrant considerably extended their mOS and mPFS in comparison to the control group (52). Based on this result, in 2019, the FDA approved the combination of apelalisb with fulvestrant for patients with advanced or metastatic breast cancer that progressed to HR+/HER2-, PIK3CA-mutated after endocrine therapy. The subsequent NEO-ORB phase II clinical study failed to demonstrate that apelalisib, in combination with letrozole, enhances neoadjuvant therapy in HR+/HER2- patients (53). The BELLE-2 and BELLE-3 studies demonstrated that buparlisib, the first pan-PI3K inhibitor to enroll in a worldwide phase III trial for HR+ MBC, extended mPFS in patients with ABC who had already advanced on treatment (54, 55). Inavolisib, as a selective inhibition of selective PI3Kα, was shown in the INAVO120 study to significantly improve mPFS in patients with locally advanced or metastatic breast cancer with PIK3CA mutation and HR+/HER2- (15.0 months vs. 7.3 months; HR, 0.43; 95% CI, 0.32–0.59; *p* < 0.001) (56). Other PI3K inhibitors, such as pictilisib and taselisib, have not delivered satisfactory therapeutic outcomes in clinical trials.

Capivasertib is a novel ATP-competitive pan-AKT kinase inhibitor that ultimately inhibits cell growth and proliferation by preventing substrate phosphorylation of AKT and thereby blocking the activation of PAM downstream targets (9). In the FAKTION phase II clinical trial, fulvestrant combined with capasertinib significantly improved mPFS in the group of HR+/HER2- ABC patients who progressed after failure of endocrine therapy compared to placebo combined with fulvestrant (10.3 months vs. 4.8 months; HR = 0.56; 95% CI 0.38–0.81; *p* = 0.0023) (57). Similarly, in the Phase III CAPItello-291 trial involving HR+/HER2- ABC patients experiencing relapse or progression during or after endocrine therapy (with or without prior CDK4/6 inhibitor therapy), the mPFS was significantly longer for those receiving capivasertib in combination with fulvestrant compared to those receiving a placebo with fulvestrant (7.2 months vs. 3.6 months; HR = 0.60; 95% CI 0.38–0.81; *p* = 0.0023; HR = 0.60; 95% CI 0.51–0.71; *p* < 0.001) (58). Capivasertib and fulvestrant have been approved by the FDA to treat patients with HR+/HER2-metastasized or locally advanced breast cancer. However, another AKT inhibitor, ipatasertib, is presently undergoing additional clinical research after failing to show a therapeutic benefit in the IPATunity130 clinical trial.

The mTOR-selective inhibitor everolimus reduces tumor proliferation by irreversibly inhibiting the phosphorylation of S6K1. In the GINECO Phase III trial, adding an mTOR inhibitor to tamoxifen alone increased the median time to progression (TTP) from 4.5 to 8.6 months in patients with HR+ ABC who had previously progressed on endocrine therapy. Similarly, in the BOLERO-2 Phase III trial, the addition of everolimus resulted in an increase in mPF from 3.2 to 7.8 months in patients with HR + ABC that had previously progressed on endocrine therapy, compared to exemestane alone (59, 60). Dual inhibition of mTORC1/2, which is currently under development, can block both S6K1, which is dependent on mTORC1 phosphorylation, and AKT, which is dependent on mTORC2 phosphorylation, thereby blocking the PAM pathway more completely (61). However, it has not yet been demonstrated that mTORC1/2 dual inhibition provides a better clinical survival benefit in breast cancer treatment compared with mTORC1 inhibitors alone (Table 2).

**Table 2:** Clinical studies of PAM signaling pathway inhibitors.

Drug	Trial	Stage	Programmatic	Patients/(numbers)	Dosage (mg)	Outcome			Reference
						mOS (months)	mPFS (months)	Others	
Alpelisib	SOLAR-1	III	alpelisib plus fulvestrant VS fulvestrant	HR+/HER2-ABC with PIK3CA-mutated (*n* = 341)	300 + 500 VS 500	39.3 VS 31.4	11.0 VS 5.7	–	52
	NEO-ORB	II	alpelisib plus letrozolt VS letrozol	HR+/HER2-,T1-T3 with PIK3CA-mutated postmenopausal women (*n* = 257)	300 + 2.5VS2.5	31.0 months 39.3 months	4.6 months 11.0 months	Not improve response in patients with HR+ eBC	53
Buparlisib	BELLE-2	III	buparlisib plus fulvestrant VS fulvestrant	HR+/HER2- ABC postmenopausal women (*n* = 1,147)	100 + 500 VS 500	–	6·9 VS 5·0	–	54
	BELLE-3	III	buparlisib plus fulvestrant VS fulvestrant	HR+/HER2- ABC postmenopausal women with progressing on or after mTOR inhibition (*n* = 432)	100 + 500 VS 500	–	3·9 VS 1·8	–	55
Inavolisib	INAVO120	III	inavolisib plus fulvestrant VS fulvestrant	HR+/HER2- ABC with PIK3CA-mutated (*n* = 341)	9 + 500 VS 500	–	15.0 VS 7.3	–	56
Capivasertib	FAKTION	II	capivasertib plus fulvestrant VS fulvestrant	HR+/HER2- ABC with ET (*n* = 183)	400 + 500 VS 500	–	10·3 VS 4·8	–	57
Everolimus	GINECO	II	everolimus plus tamoxifen VS tamoxifen	HR+/HER2-,AI-resistant MBC (*n* = 111)	10 + 20 VS 20	–	–	median TTP 8.6 VS 4.5	59
	BOLERO-2	III	everolimus plus exemestane VS exemestane	HR+/HER2- ABC with ET (*n* = 724)	10 + 25 VS 25	31.0 VS 26.6	7.8 vs. 3.2	–	60

ET, endocrine therapy; ABC, advanced breast cancer; MBC, metastatic breast cancer; eBC, early breast cancer; TTP, time to progression.

## 4 Targeting the HER2 signaling pathway

### 4.1 HER2 signaling pathway in HR+ breast cancer

**Figure 3:**
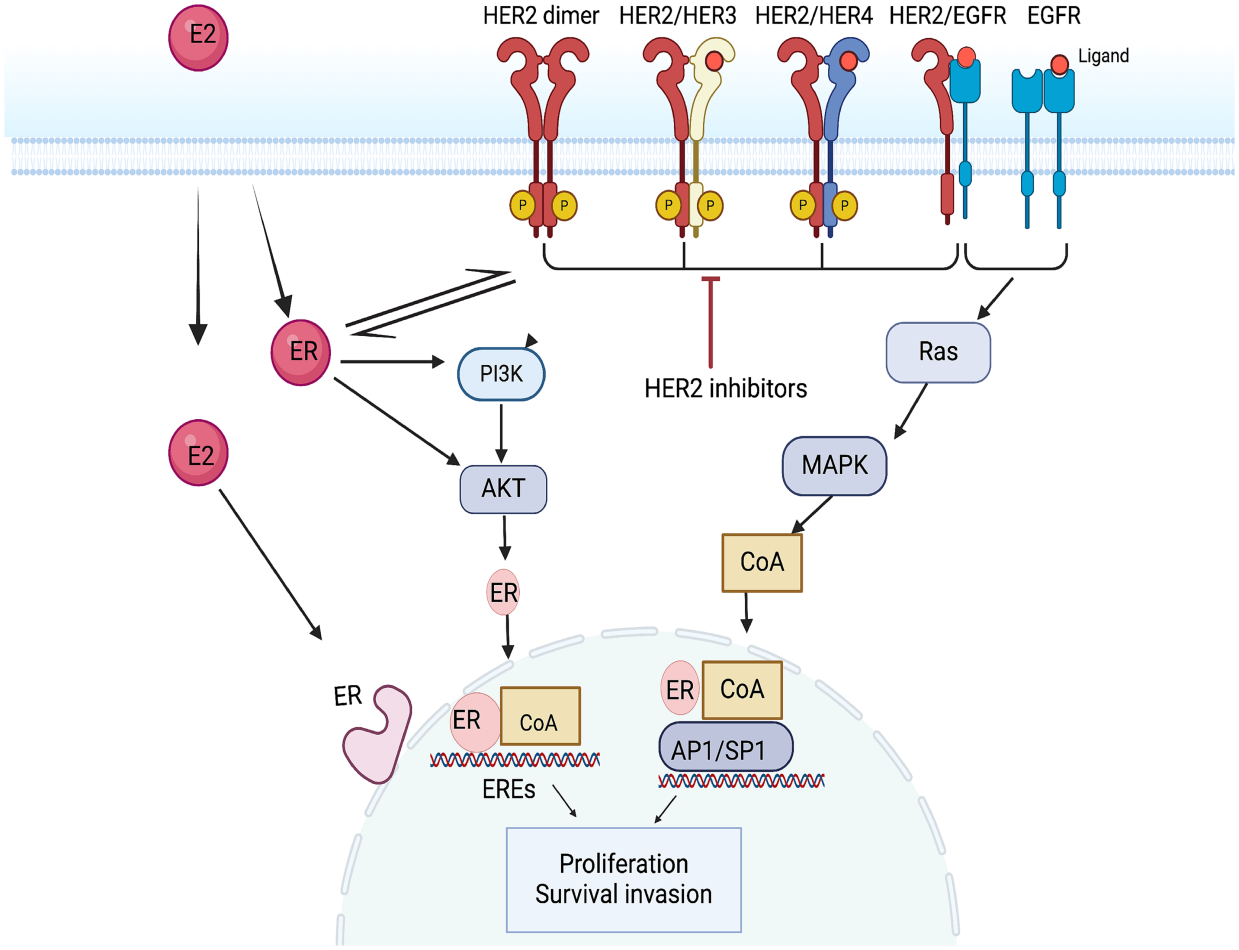
Mechanism of action of the HER2 pathway and anti-HER2 targets. EREs, ER exit sites; AP1, activator protein 1; SP1, transcription Specificity Protein 1.

HER2-positive (HER2+) breast cancers represent about 10% of all breast cancer cases. This subtype is also referred to as luminal B2, characterized not only by estrogen activation but also by the overexpression of HER2 and the interaction between these two signaling pathways (62, 63). Estrogen receptors (ER) activate non-nuclear and non-genomic pathways through their interaction with receptor tyrosine kinases, such as HER2, and their downstream signaling intermediates, including p42/44 mitogen-activated protein kinase (MAPK) and Akt. This interaction leads to increased cell proliferation. At the same time, overactive HER2 signaling activates downstream kinases, including Akt and MAPK, which can reduce the expression of ER at both the mRNA and protein levels. Nonetheless, these kinases also phosphorylate ER and its co-regulators, thereby enhancing and regulating ER’s transcriptional activity. This phenomenon can counteract the effects of endocrine therapy and contribute to endocrine resistance (64) (Figure 3). According to the guidelines from the American Society of Clinical Oncology (ASCO), adding anti-HER2 targeted therapy is more effective for this specific group of HER2+ patients (Figure 3).

### 4.2 HER2 low expression and ultra low expression

In recent years, differences observed in HER2- mid-tests have led to new definitions of HER2-. Specifically, cases exhibiting immunohistochemistry (IHC) scores of 1+ or 2+, but which are FISH-negative, are now categorized as HER2 hyperexpression. Meanwhile, the presence of faint or barely detectable incomplete staining in less than 10% of tumor cells, which is not amplified by FISH examination, is classified as HER2 ultralow expression (65, 66). Although HER2 underexpression and ultralow expression do not significantly contribute to the proliferation and differentiation of breast cancer cells, there is increasing evidence that targeting HER2 therapy can still provide clinical benefits in cases of underexpression and ultralow expression.

### 4.3 Anti-HER2 drugs in HR+/HER2 + BC

Trastuzumab and patuzumab are recombinant humanized monoclonal antibodies that target HER2 to prevent cancer growth, and the TANDEM, EGF30008, and eLEcTRA studies have demonstrated the value of single-targeted endocrine therapies (67–69). However, there remains a risk of recurrence with single-agent targeted therapy. As a result, clinical research is now shifting its focus toward dual-targeted chemotherapy (70). The APHINITY and PERTAIN studies have demonstrated that a combination of dual-targeted endocrine or chemotherapy provides greater clinical benefits than single-targeted therapies alone (71, 72).

Pyrotinib is an irreversible pan-HER2 tyrosine kinase inhibitor (TKI) targeting HER1, HER2, and HER4, and a multicenter, single-arm, phase II clinical trial initially demonstrated that treatment with pyrotinib in combination with letrozole could be used as first-line therapy in patients with HR+/HER2- MBC (73). In another multicenter, single-arm, phase II clinical trial, pyrotinib in combination with fulvestrant increased mPFS in HR+/HER2- MBC patients who had failed prior trastuzumab therapy, suggesting that pyrotinib in combination with fulvestrant may be a practical approach (74). Neratinib binds primarily to EGFR (HER1) and HER4, thereby blocking signaling. The ExteNET study demonstrated a significant reduction in the risk of disease recurrence when neratinib was initiated within 1 year of trastuzumab-based therapy in a population of patients with treated HER2+/HR+ BC (75). Lapatinib, a selective oral tyrosine kinase inhibitor (TKI), was evaluated in the NeoALTTO study, which found that combining lapatinib with trastuzumab increased the complete remission rate in patients with HER2+ eBC. However, no long-term benefits were observed in subsequent follow-ups (76). Tucatinib, in combination with trastuzumab and capecitabine, significantly reduced the risk of intracranial progression and the risk of death in patients with brain metastases from HER2 + BC who were already receiving treatment in the HER2CLIMB trial (77).

Antibody-drug conjugates (ADCs) are monoclonal antibodies that are covalently linked to biologically active cytotoxic drugs through chemical linkers. This design allows them to specifically target tumor tissues and release cytotoxic agents, enhancing therapeutic efficacy while minimizing adverse effects (78–80). Enmetrastuzumab (T-DM1) was the first FDA-approved drug for the treatment of HER2+ MBC and ADCs that did not achieve pathologic complete remission (pCR) after neoadjuvant therapy. In the EMILIA study, T-DM1 was shown to prolong mPFS and OS compared to capecitabine combined with lapatinib in patients with HER2+ MBC (81). In the KATHERINE study, patients with eBC who did not achieve a pCR after neoadjuvant therapy for HER2+ achieved longer PFS with enmetrastuzumab compared to receiving trastuzumab monotherapy (82). Trastuzumab deruxtecan (T-Dxd) has exhibited durable antitumor activity in the DESTINY-Breast01 clinical trial involving previously treated HER2-positive MBC patients. However, in the DESTINY-Breast03 trial, T-Dxd did not show a significant improvement in PFS compared to T-DM1 for previously treated HER2-positive MBC patients receiving trastuzumab and paclitaxel analogs, although it did result in a notable increase in mPFS for patients with HER2-positive breast cancer (83).

### 4.4 Anti-HER2 drugs in HER2-low and HER2-ultralow breast cancer

Trastuzumab has been validated as the first HER2-targeted agent that improves outcomes for patients with HER2-low or metastatic breast cancer. In the DESTINY-Breast04 trial, it significantly prolonged the survival of patients with HER2-overexpressing metastatic breast cancer who had previously received chemotherapy, regardless of HR status, when compared to chemotherapy alone (84). DESTINY-Breast06, on the other hand, further demonstrated the role of T-Dxd in patients with HER2 low or HER2 -ultralow /HR+ ABC (85). In addition, the PILHLE-001 trial demonstrated a lower residual cancer burden with neoadjuvant pyrotinib in combination with chemotherapy in HER2- high-risk EBC (86) (Table 3).

**Table 3:** Clinical studies of anti-HER2 drugs.

Drug	Trial	Stage	Programmatic	Patients/(numbers)	Dosage (mg)	Outcome			Reference
						mOS (months)	mPFS (months)	Others	
Trastuzumab	TAnDEM	III	trastuzumab plusanastrozole versus anastrozole	HR+/HER2+ MBC (*n* = 207)	4 mg/kg on day 1, 2 mg/kg every week+1 VS 1	–	4.8 VS 2.4	–	67
	eLEcTRA	III	trastuzumab plus letrozole VS letrozole	HR+/HER2+ MBC (*n* = 93)	4 mg/kg on day 1, 2 mg/kg every week+2.5 VS 2.5	–	–	Median TTP 14.1 VS 3.3	69
Pertuzumab	APHINITY	III	pertuzumab plus trastuzumab and AI VS trastuzumab plus AI	Early HER2+ BC (*n* = 4,805)	840 mg loading dose followed by 420 mg every 3 weeks plus 8 mg/kg followed by 6 mg/kg every 3 weeks	–	–	OS: 95% VS 94% IDFS: 91% VS 88%	71
	PERTAIN	II	pertuzumab plus trastuzumab and AI VS trastuzumab plus AI	HR+/HER2+ MBC/LABC (*n* = 129)	840 mg loading dose followed by 420 mg every 3 weeks plus 8 mg/kg followed by 6 mg/kg every 3 weeks and 1 /2.5 VS 8 mg/kg followed by 6 mg/kg every 3 weeks and 1 /2.5	–	18.89 VS 15.80	–	72
Lapatinib	EGF30008	III	lapatinib plus letrozole VS letrozole	postmenopausal women with HR+/HER2+ MBC (*n* = 1,286)	1,500 + 2.5 VS 2.5	33.0 VS 32.3	8.2 VS 3.0	ORR 28% vs. 15%	68
Pyrotinib	PLEHERM	II	pyrotinib plus letrozole	HR+/HER2+ MBC (*n* = 53)	400 + 2.5	–	13.7	–	73
	NCT04034589	II	pyrotinib plus fulvestrant	HR+/HER2+ MBC with trastuzumab treatment before (*n* = 46)	400 + 500(on days 1 and 15 of cycle 1, then on day 1 of each subsequent monthly cycle)	–	18.2	–	74
	PILHLE-001	II	pyrotinib puls chemotherapy	luminal/HER2-low eBC (*n* = 49)	unreported	–	–	Encouraging efficacy and manageable toxicity	86
Neratinib	ExteNET	III	neratinib VS placebo	HR+/HER2+ eBC with neoadjuvant/adjuvant trastuzumab-based therapy (*n* = 2,840)	240 VS placebo	–	–	5 years iDFS: 93.0%VS 91.7%	75
Lapatinib	NeoALTTO	III	lapatinib plus trastuzumab VS lapatinib VS trastuzumab	HER2+ eBC (*n* = 455)	1,000+ 4 mg/kg(subsequent doses 2 mg/kg) VS 4 mg/kg(subsequent doses 2 mg/kg) VS 1500	–	–	pCR: 51·3%VS 29·5% VS 24·7%	76
Tucatinib	HER2CLIMB	III	tucatinib plus trastuzumab and capecitabine VS trastuzumab and capecitabine	HER2+ MBC/LABC (*n* = 129)	300 + 6 mg/kg(subsequent doses 8 mg/kg) + 1,000 mg/m^2^ VS 6 mg/kg(subsequent doses 8 mg/kg) + 1,000 mg/m^2^	24.7 VS 19.2	7.6 VS 4.9	–	77
T-DM1	EMILIA	III	tucatinib VS capecitabine plus lapatinib	HER2+ MBC/LABC treated with trastuzumab and a taxane before (*n* = 991)	3·6 mg/kg(every 3 weeks) VS 1000 mg/m^2^(orally twice daily on days 1–14 on each 21-day cycle) + 1,250 mg(once daily on days 1–21)	29·9 VS 25·9	29·9 VS 25.9	–	81
	KATHERINE	III	T-DM1 VS trastuzumab	HER2+ BC with neoadjuvant therapy before (*n* = 1,486)	3.6 mg/kg(every 3 weeks for 14 cycle) VS 6 mg/kg(every 3 weeks for 14 cycles)	–	–	iDFS: 88.3% VS 77.0%	82
T-Dxd	DESTINY-Breast03	III	T-Dxd VS T-DM1	HER2+ MBC (*n* = 524)	5·4 mg/kg VS 3·6 mg/kg	–	26·5 VS 6·8	–	83
	DESTINY-Breast04	III	T-Dxd VS physician’s choice of chemotherapy	HER2-low MBC with one or two previous lines of chemotherapy before (*n* = 557)	5·4 mg/kg VS chemotherapy	23.4 VS 16.8	9.9 VS 5.1	–	84
	DESTINY-Breast06	III	T-Dxd VS physician’s choice of chemotherapy	MBC(involving low and ultralow HER2 expression) with ET before (*n* = 866)	5·4 mg/kg VS chemotherapy	–	13.2 VS 8.1	–	85

MBC/LABC, metastatic/locally advanced breast cancer; AI, aromatase inhibitor; pCR, pathologic complete response; iDFS, invasive disease-free.

## 5 Targeting the tumor microenvironment

### 5.1 Tumor microenvironment of breast cancer

Tumor cells, immune cells, fibroblasts, cytokines released by stromal and related cells, and non-cellular elements like microvessels make up the complex ecosystem known as the tumor microenvironment. These elements are essential for controlling the fundamental survival and sustaining the function of tumor cells (87). Different solid tumors have distinct tumor microenvironment compositions, and breast cancer usually has large concentrations of immunosuppressive cells and cancer-associated fibroblasts (CAF) (88). Furthermore, different breast cancer subtypes have different tumor microenvironments; HR+ breast cancers, in contrast to triple-negative and HER2+ breast cancers, show lesser immune cell infiltration and a worse response to immune checkpoint blockade (76). Potential targets for tumor therapy have been investigated in recent attempts to target the tumor microenvironment. Vascular endothelial growth factor and fibroblast growth factor are the two targets of this review.

### 5.2 Vascular endothelial growth factor inhibitors in breast cancer

**Figure 4:**
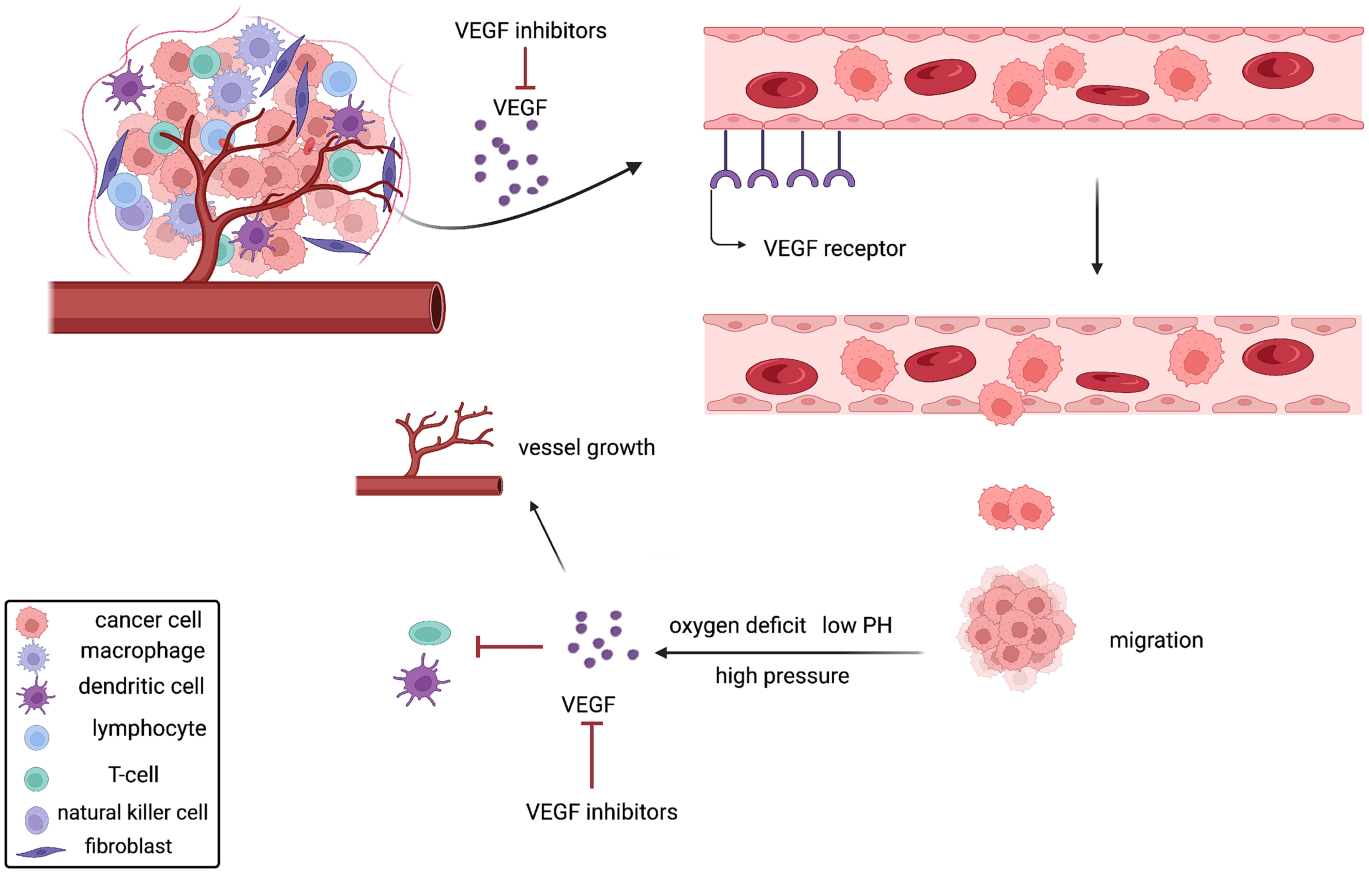
Mechanism of action of VEGF inhibitors.

Angiogenesis plays a critical role in the formation and dissemination of tumors by supplying oxygen and nutrients to the tumor, which promotes tumor growth. Vascular endothelial growth factor (VEGF) is produced in response to the tumor’s high pressure, low oxygen content, and low pH, which encourages the development of neovascularization. In order to facilitate tumor growth and metastasis, these new blood vessels typically lose their natural structure, take on a convoluted shape, and have increased permeability. Additionally, VEGF suppresses dendritic cell maturation and antigen presentation by recruiting and inhibiting anti-tumorigenic regulatory T cells by chemotactic action (89) (Figure 4). Interestingly, a separate study found that the size of big arteries in the tumor vasculature was likewise linked to a lower survival rate in HR+ breast cancer (90). Thus, inhibition of vascular growth is considered to have antitumor effects in HR+ BC.

Bevacizumab is an anti-VEGF monoclonal antibody that the FDA currently approves for the treatment of advanced metastatic breast cancer. In the phase III clinical trial (NCT00333775), the combination of bevacizumab and docetaxel as a first-line treatment for HER2-negative, locally recurrent, or metastatic breast cancer demonstrated a slight improvement in mPFS compared to the chemotherapy-only group however, no benefit in overall survival OS was observed (91). In the RIBBON-2 clinical trial, bevacizumab was used as a second-line treatment for patients with HER2-positive metastatic breast cancer. This treatment increased the mPFS from 5.1 months to 7.2 months (HR, 0.78; 95% CI, 0.64 to 0.93; *p* = 0.0072) compared to chemotherapy alone. However, no OS benefit was observed (92). Bevacizumab, in combination with NCT, increased the rate of histological remission in HER2-negative breast cancer in the NCT00567554 clinical study (93). Additionally, the ROSE/TRIO-12 clinical study investigated the use of docetaxel in combination with ramucirumab, another anti-vascular growth factor inhibitor, instead of using docetaxel alone. The study found that ramucirumab did not improve the mPFS in the first-line therapy of metastatic breast cancer (94). Although several phase II clinical trials have been carried out, neither monotherapy nor combination therapy has been shown to improve survival thus far (95, 96).

### 5.3 Fibroblast growth factor inhibitors in breast cancer

The FGF/FGFR signaling pathway, which consists of fibroblast growth factor receptors (FGFR) and fibroblast growth factors (FGF), plays a crucial role in regulating cell proliferation, survival, migration, and differentiation (97, 98). Abnormalities in this pathway are frequently linked to the development and progression of cancer, as well as drug resistance. There are four subtypes of FGFRs: FGFR1, FGFR2, FGFR3, and FGFR4. Research has shown that FGFR1 amplification occurs in approximately 15% of patients with estrogen receptor-positive breast cancer. Consequently, targeting and inhibiting FGFR could be an effective treatment strategy for this type of cancer (99, 100).

Dovitinib is a multi-targeted oral tyrosine kinase inhibitor that effectively inhibits the proliferation of breast cancer cell lines that have amplified FGFR1/2. However, it does not impact cell lines with normal FGFR. In a phase II clinical study (NCT01528345), dovitinib, when combined with fulvestrant, demonstrated promising clinical efficacy for the treatment of postmenopausal breast cancer patients who were HR+ and HER2- and who had progressed despite prior ET. Unfortunately, the clinical trial has been suspended due to issues with the selection of the inclusion group (101). To better target the FGF/FGFR signaling pathway, selective FGFR inhibitors (infigratinib, erdafitinib, AZD4547, Debio1347, TAS-120) have been developed. In a phase II trial (NCT01795768), the FGFR1/2/3 inhibitor AZD4547 demonstrated potential therapeutic effects in patients with FGFR1-amplified breast tumors (102) (Table 4).

**Table 4:** Clinical studies of VEGF inhibitors and FGFR inhibitors.

Drug	Trial	Stage	Programmatic	Patients/(numbers)	Dosage (mg)	Outcome			Reference
						mOS (months)	mPFS (months)	Others	
Bevacizumab	NCT00333775	III	bevacizumab puls docetaxel VS docetaxel	HER2- MBC (*n* = 736)	7.5/15 mg/kg + 100 mg/m^2^ (every 3 weeks) VS 100 mg/m^2^ (every 3 weeks)	–	10.1 (15 mg/kg) VS 8.2	–	91
	RIBBON-2	II	bevacizumab puls chemotherapy VS chemotherapy	HER2- MBC (*n* = 684)	15 mg/kg (every 3 weeks) or 10 mg/kg (every 2 weeks) VS chemotherapy	–	7.2 VS 5.1	–	92
	NCT00567554	II	bevacizumab puls NACT VS NACT	HER2- eBC (*n* = 1948)	15 mg/kg(8 cycle) + NACT VS NACT	–	-	pCR: 18.4% VS 14.9%	93
Ramucirumab	ROSE/TRIO-12	III	ramucirumab plus docetaxel VS docetaxel	HER2- MBC (*n* = 1,144)	10 mg/kg every 3 weeks+75 mg/m^2^ VS 75 mg/m^2^	27.3 VS 27.2	9.5 VS 8.2	–	94
Dovitinib	NCT01528345	II	dovitinib puls fulvestrant VS fulvestrant	HR^+^/HER2^−^ MBC/LABC (*n* = 97)	500+ 500 mg (every 4 weeks, then every 2 weeks) VS 500 mg (every 4 weeks, then every 2 weeks)	–	5.5 VS 5.5	–	101

ABC, advanced breast cancer; eBC, early breast cancer; MBC, metastatic breast cancer; MBC/LABC, metastatic/locally advanced breast cancer; pCR, pathologic complete response.

## 6 Targeting BRCA gene mutations

**Figure 5:**
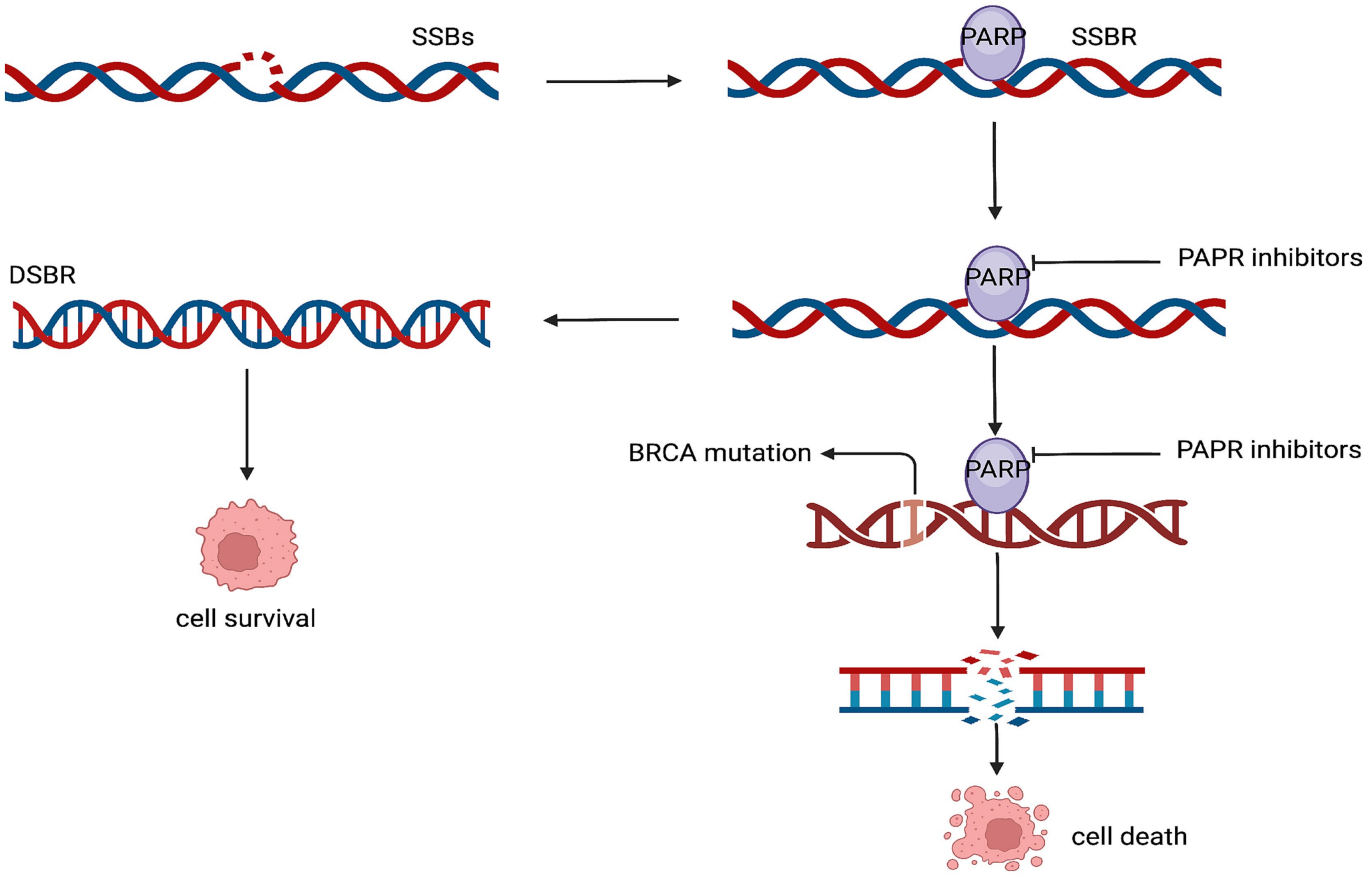
Mechanism of action of the PARP inhibitors. SSBs, single-strand breaks; SSBR, single-strand break repair; DSBR, double-strand break repair.

The homologous recombination repair pathway uses the protein encoded by the BRCA gene to repair DNA double-strand breaks. Germline mutations in the BRCA gene are currently present in approximately 5% of patients with breast cancer. While patients with BRCA2 mutations usually have tumors that exhibit estrogen receptors, those with BRCA1 mutations are more likely to develop triple-negative breast cancer. Furthermore, poly (ADP-ribose) polymerase (PARP) is essential for fixing single-strand breaks in DNA (103, 104). The application of PARP inhibitors in patients with BRCA mutations results in an antitumor effect by causing apoptosis of the tumor cells due to the inability to repair both the single and double-strand (105) (Figure 5).

Olaparib, the first FDA-approved PARP inhibitor, has been approved as an adjuvant treatment option for HR+/HER2- ABC with germline BRCA1 or BRCA2 mutations or early-stage, high-risk breast cancer that has received neoadjuvant or adjuvant chemotherapy. In the OlympiAD clinical trial, patients with BRCA-mutated and HER2- MBC treated with olaparib had significantly longer mPFS than the standard treatment group (106). A randomized, double-blind, phase III clinical trial (NCT02849496) demonstrated that adding olaparib to neoadjuvant or adjuvant chemotherapy significantly improved IDFS in patients with early-stage HER2- breast cancer who also had a BRCA1 or BRCA2 mutation. The IDFS rates were 87.5% for those receiving olaparib in combination with chemotherapy compared to 80.4% for those receiving chemotherapy alone (107). Talazoparib, the second FDA-approved PARP inhibitor for the treatment of advanced metastatic breast cancer, improved mPFS in patients with gBRCA-mutated, locally advanced/metastatic BC in combination with chemotherapy compared to chemotherapy alone in the EMBRACA clinical trial (108). Other PARP inhibitors have not yet been tested in phase III clinical trials, and niraparib, in combination with pembrolizumab, showed promising antitumor activity in a phase II clinical trial (NCT02657889) (109) (Table 5).

**Table 5:** Clinical studies of PARP inhibitors and HDAC inhibitors.

Drug	Trial	Stage	Programmatic	Patients/(numbers)	Dosage (mg)	Outcome			Reference
						mOS (months)	mPFS (months)	Others	
Olaparib	OlympiAD	III	olaparib VS TPC	HER2^−^ MBC with BRCA mutation (*n* = 302)	600 mg VS TPC	19.3 VS 17.1	18.9 VS 15.5	–	106
	NCT02849496	III	olaparib VS placebo	HER2^−^ eBC with BRCA mutation and had finished local treatment and neoadjuvant or adjuvant chemotherapy (*n* = 1836)	300 VS placebo	–	–	iDFS: 87.5%, VS 80.4%	107
Talazoparib	EMBRACA	III	talazoparib VS PCT	ABC with BRCA1/2 mutation (*n* = 431)	1 VS PCT	–	8.6 VS 5.6	ORR:62.6% VS 27.2%	108
Tucidinostat	ACE	III	tucidinostat plus exemestane VS exemestane	HR + ABC with postmenopausal woman (*n* = 365)	30 (twice a week) + 25 VS 25	–	7·4 VS 3·8	–	113
Entinostat	ENCORE301	II	entinostat plus exemestane VS exemestane	HR + ABC with postmenopausal woman (*n* = 130)	5 (once a week) + 25 VS 25	28.1 VS 19.8	4.3 VS 2.3	–	114

ABC, advanced breast cancer; eBC, early breast cancer; iDFS, invasive disease-free; ORR, overall response rates.

## 7 Targeting HDAC

### 7.1 HDAC in breast cancer

**Figure 6:**
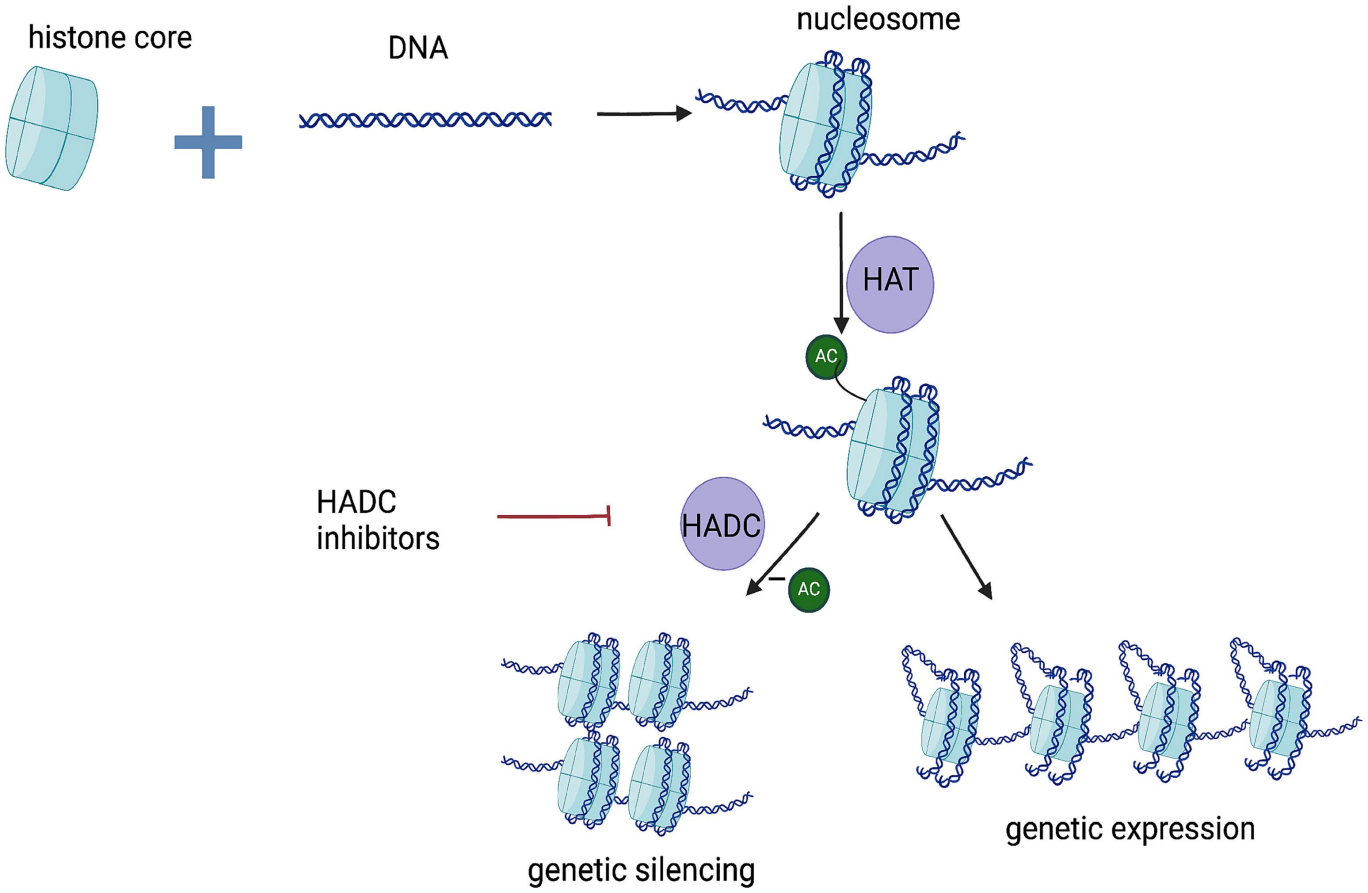
Mechanism of action of the HADC inhibitors. Ac, acety.

Cancer is a genetic and epigenetic disease. Epigenetic inheritance refers to heritable changes in gene expression or cellular expression caused by various modifications to DNA, such as methylation, acetylation, and chromatin remodeling, without altering the DNA sequence. Histone acetyltransferases (HAT) acetylate histones, which results in more open or loose chromatin structure causing gene expression. Histone deacetylases remove acetyl groups from histones, favoring chromatin compaction. Usually associated with gene silencing (110, 111). By inhibiting HDAC, histones remain acetylated, which allows for the reactivation of silenced anti-oncogenes and promotes tumor apoptosis (Figure 6). Additionally, HDAC is a key regulator of various tumor-related physiological processes, including angiogenesis, cell cycle regulation, immune response, DNA repair, and apoptosis. Therefore, blocking HDAC can inhibit tumor growth by targeting multiple pathways.

### 7.2 HDAC inhibitors

Histone deacetylases (HDACs) are categorized into four main classes: I, II, III, and IV. Cell nuclei are where Classes I and II mostly operate. In contrast, class III HDACs, also known as sirtuins, require NAD+ and play a role in a broader range of cellular processes, including metabolism and cell aging (senescence). Class IV HDACs are predominantly expressed in the brain, heart, testis, and kidneys.

Tucidinostat is a subtype-selective histone deacetylase (HDAC) inhibitor that explicitly inhibits Class I and II (112). In the ACE clinical trial, treatment with tucidinostat in combination with exemestane significantly improved patient prognosis and slowed disease progression compared with exemestane alone in postmenopausal endocrine-resistant advanced HR+ breast cancer (113).

Entinostat, a selective inhibitor of HDAC I and IV, showed a significant improvement in mPFS and mOS in the phase II (ENCORE301) trial of exemestane plus entinostat in HR+ ABC patients who had progressed on prior endocrine therapy alone, compared with the exemestane alone group (114). Entinostat in combination with exemestane did not result in a survival benefit for breast cancer patients in the subsequent phase III clinical study of E2112 (115) (Table 5).

## 8 Summary and outlook

Breast cancer is the most prevalent malignant tumor in women and is a serious health risk to them. About 70–75% of BC are HR+, the most prevalent subtype. Even though hormone therapy works well for these patients, the condition typically worsens over time. Furthermore, endocrine therapy by itself is unable to satisfy the clinical requirements of advanced or high-risk patients, and the harmful side effects of chemotherapy deter some patients. Recent advancements in targeted therapies have transformed treatment approaches for HR+ breast cancer patients.

Numerous clinical trials have confirmed the efficacy of CDK4/6is as a first-line treatment for HR+/HER2- ABC, which has greatly improved patient outcomes. Clinical trials supporting the use of CDK 4/is in adjuvant early therapy, neoadjuvant endocrine therapy, and advanced therapy for patients who have had prior treatments have shown encouraging results. Nevertheless, several researchers have pointed up shortcomings in these clinical studies, citing problems like data processing, treatment dosage selection, and imbalances in baseline characteristics. These elements might have had an impact on how consistently the favorable outcomes were seen (116). However, CDK4/6is resistance should not be disregarded. Since the resistance route may be linked to Rb1 deletion, over-activation of CDK2, and the PIK3CA/Akt/mTOR pathway, it is currently challenging to reduce CDK4/6is resistance in order to extend the drug’s effectiveness.

PI3K/AKT/mTOR pathway-targeted medicines are presently employed as second-line therapy for HR+ MBC, primarily in patients with breast cancer who have had prior CDK4/6is progress. Although additional clinical evidence is required to support this, some clinicians have also suggested PAM pathway inhibitors as first-line treatment for endocrine-resistant individuals who have mutations in the PAM pathway. Significant obstacles in medication therapy also include resistance to PAM system inhibitors and adverse responses such inhibitor-associated hyperglycemia and hyperinsulinemia, stomatitis, and skin rashes brought on by PAM pathway inhibition (117). Moreover, the effects of PAM pathway inhibitors alone are limited and usually combined with other drugs and therapies. A direction that needs to be developed is how to perform drug combinations to reduce side effects and improve therapeutic efficacy. Meanwhile, the development of biomarkers for PAM inhibitors is challenged by the complex interactions of the pathway, which makes it difficult to detect the efficacy of the drugs.

Further exploration of molecular typing has led to further delineation of HER2- and clinical trials have demonstrated that patients with HER2 low-expressing breast cancers may derive more benefit from anti-HER2 ADCs and that HER2-ultralow expression also benefits from anti-HER2, which would provide new therapeutic options in clinical practice. However, there is still controversy about the boundary between HER2-zero and HER2-ultralow and questions about the mechanism of action leading to the antitumor activity of anti-HER2 drugs in HER2-low breast cancers, perhaps related to the targeted delivery of cytotoxic molecules of ADC drugs, but not to the blockade of the HER2 pathway make the concept of HER2 low- and ultralow-expression limited application in the clinical setting (118).

The tumor microenvironment is the soil that promotes tumor progression and metastasis, and inhibiting angiogenesis in the microenvironment is thought to be an effective way to combat tumor metastasis. But it is important to remember, as well, that prolonged use of anti-angiogenic medications can increase tumor invasiveness, strengthen the hypoxic response, and decrease drug transport, all of which can result in drug resistance and even cancer metastases (119). Moreover, unlike what was initially envisioned as fewer adverse events due to the targeted nature of the drugs, anti-angiogenic drugs are also prone to common serious adverse events such as hypertension, proteinuria, and bone marrow suppression. For the development of specific biomarkers, some studies have been reported to confirm that the size of tumor blood vessels can be detected as a biomarker in HR+ breast cancer. However, specific biomarkers need to be explored more deeply due to the complexity of angiogenic pathways, tumor heterogeneity, and many other factors.

PARP inhibition has shown great potential in cancer therapy, especially in tumors with breast cancer susceptibility gene mutations and other DNA repair defects. However, drug resistance is currently a significant challenge. Resistance may be related to the recovery of homologous recombination repair (HRR), overexpression of ATP-binding cassette drug transporter proteins, and stable replication forks. It has been shown that the therapeutic efficacy can be optimized by combining it with other therapeutic approaches.

Targeted epigenetics have been shown to inhibit tumor growth and progression. They are currently showing good efficacy in breast cancer, similarly limiting their application because of toxic side effects, tumor heterogeneity, and off-target effects.

In conclusion, patients with HR+ breast cancer now have more therapeutic options thanks to the ongoing development of targeted medicines. Clinical trials have shown promising therapeutic results for these medications. Their use in clinical practice has been constrained, nevertheless, by severe side effects, off-target effects, resistance to targeted medications, and a lack of accurate and precise biomarkers. The use of drug combinations to overcome side effects and drug resistance, the investigation of more precise drug delivery methods and targeted targets to minimize off-target effects and enhance therapeutic efficacy, and the discovery of more precise biomarkers to gage therapeutic efficacy are some of the future development trends. Clinicians and pathologists should focus on developing more effective, less harmful, and customized treatments for patients as molecular typing continues to be explored and improved.
